# Patient and public involvement of young people with a chronic condition: lessons learned and practical tips from a large participatory program

**DOI:** 10.1186/s40900-020-00234-1

**Published:** 2020-09-30

**Authors:** Femke van Schelven, Eline van der Meulen, Noortje Kroeze, Marjolijn Ketelaar, Hennie Boeije

**Affiliations:** 1 Netherlands Institute for Health Services Research, Utrecht, The Netherlands; 2 JongPIT Foundation, Amsterdam, The Netherlands; 3 Center of Excellence for Rehabilitation Medicine, UMC Utrecht Brain Centre, University Medical Centre Utrecht, and De Hoogstraat Rehabilitation, Utrecht, the Netherlands

**Keywords:** Patient participation, Involvement, Engagement, Adolescence, Child disability, Chronic disease

## Abstract

**Plain English summary:**

**Background**

Young people with a chronic condition are increasingly involved in doing research and developing tools and interventions that concern them. Working together with patients is called Patient and Public Involvement (PPI). We know from the literature that PPI with young people with a chronic condition can be challenging. Therefore, it is important that everyone shares their lessons learned from doing PPI.

**Aim**

We want to share our lessons learned from a large program, called Care and Future Prospects. This program helps young people with a chronic condition to, for example, go to school or to find a job. It funded numerous projects that could contribute to this. In all projects, project teams collaborated with young people with a chronic condition.

**What did we do**

We asked young people with a chronic condition and project teams about their experiences with PPI. Project teams wrote reports, were interviewed, and filled out a tool called the Involvement Matrix. Young people filled out a questionnaire.

**Findings**

In the article, we present our lessons learned. Examples are: it is important to involve young people with a chronic condition from the start of a project and everyone involved in a project should continuously discuss their responsibilities. We provide practical tips on how young people with a chronic condition and project teams can do this. A tip for young people is, for example: ‘discuss with the project team what you can and want to do and what you need’. An example of a tip for project teams is: ‘Take time to listen attentively to the ideas of young people’.

**Abstract:**

**Background**

The Patient and Public Involvement (PPI) of young people with a chronic condition receives increasing attention in policy and practice. This is, however, not without its challenges. Consequently, calls have been made to share lessons learned during PPI practice.

**Methods**

We share our lessons learned from a large participatory program, called Care and Future Prospects. This program aims to improve the social position of young people aged 0–25 with a physical or mental chronic condition by funding participatory projects. We have drawn our lessons from 33 of these projects, using four data sources. One data source provided information from the perspective of young people with a chronic condition, i.e. questionnaires. Three data sources contained information from the perspectives of project teams, i.e. project reports, case studies of projects and Involvement Matrices. For most of the projects, we have information from multiple data sources.

**Results**

We have combined the findings derived from all four data sources. This resulted in multiple lessons learned about PPI with young people with a chronic condition. Those lessons are divided into six themes, including practicalities to take into account at the start, involvement from the start, roles and responsibilities, support, flexibility and an open mind, and evaluation of process and outcomes.

**Conclusions**

The lessons learned have taught us that meaningful PPI requires effort, time and resources from both young people and project teams, from the beginning to the end. It is important to continuously discuss roles and responsibilities, and whether these still meet everyone’s needs and wishes. Our study adds to previous research by providing practical examples of encountered challenges and how to deal with them. Moreover, the practical tips can be a valuable aid by showing young people and project teams what concrete actions can support a successful PPI process.

## Background

The Patient and Public Involvement (PPI) of young people with a chronic condition, such as rheumatism, diabetes, cerebral palsy and traumatic brain injury, receives increasing attention in policy and practice [1, 2]. Young people with a chronic condition are increasingly invited to contribute to research projects, i.e. scientific projects aimed at increasing knowledge, and innovation projects, i.e. practice-oriented projects aimed at developing, for example, tools and interventions. This development is driven by the realisation that involving those young people as *co-actors* in decision-making processes contributes to the relevance of projects [3–5] and improves the uptake of results [4, 6, 7]. According to the United Nations Convention on the Rights of the Child, it is a fundamental right of young people to have a say in matters that affect them, without discrimination and irrespective of disabilities [8].

An early definition of young people’s PPI has been provided by Hart [9]. He described it as “the process of sharing decisions which affect one’s life and the life of the community in which one lives”. Based on work of Arnstein on citizen participation [10], he developed a Ladder of Involvement which distinguishes eight roles that provide young people with different degrees of agency when working together with adults. Although a lot of thinking about PPI is still based on and inspired by these principles of Hart’s model, it has also been criticised [11–13]. Especially the hierarchy implied by the ladder and the elements of tokenism included in the model are topics of discussion. Consequently, several researchers have adapted the model and/or formulated new definitions [12, 14]. Nowadays, the INVOLVE’s definition is one that is often referred to: PPI is “research [or innovation projects] being carried out *with* or *by* members of the public rather than *to*, *about* or *for* them” [1].

Despite the realisation that the perspectives of young people with a chronic condition should not be neglected in research and innovation projects addressing issues that concern them, many young people and project teams are struggling how to shape their collaboration. Substantive issues include, for example, how to achieve meaningful impact on the relevance and quality of research and innovation projects [6, 15] and how to handle differences in ideas and opinions [16, 17]. More practical challenges were observed as well. PPI of young people with a chronic condition requires an additional effort, in terms of time and resources, from both project teams and young people with a chronic condition [3, 4, 6, 7, 18, 19]. Also, questions remain about what roles young people with a chronic condition can play and in what project phases [6, 11, 19, 20]. The health issues, fatigue and sudden hospital stays young people with a chronic condition have to cope with can make a PPI process even more complicated.

Limited progress has been made in addressing the challenges of PPI of young people with a chronic condition in recent years. Project teams have encountered similar challenges and reported on them time and again [2]. Failure to overcome these challenges can cause insecurity and a fear of doing it wrong among both young people with a chronic condition and project teams, discouraging future attempts of PPI [21]. In this light, several calls have been made to share experiences and lessons learned on PPI and on how to deal with its challenges [22, 23].

In this article, we wish to respond to these calls and share our lessons learned about PPI with young people with a chronic condition from a four-year participatory program, called Care and Future Prospects [in Dutch: Zorg én Perspectief] (CFP). CFP aimed to improve the social position of young people aged 0–25 with a chronic condition, i.e. conditions that ‘that lasted or are expected to last twelve or more months and resulted in functional limitations and/or the need for ongoing medical care’ [24]. In order to achieve, CFP funded numerous participatory research and innovation projects (Table 1). In an evaluation study conducted between 2016 and 2019, we investigated whether they had achieved this goal. The data collected included valuable lessons about PPI of young people with a chronic condition in research and innovation projects. In this article, we aim to share these lessons and provide practical tips for young people and project teams, by answering the following research questions:

- How have young people with a chronic condition been involved in research and innovation projects in the large participatory program of CFP?
- What difficulties do young people with a chronic condition and project teams encounter during their collaboration?
- What are conditions for meaningful PPI, both for young people with a chronic condition and project teams?
- What impact of PPI do young people with a chronic condition and project teams experience?

**Table 1:** The Care and Future Prospects Program

The Dutch organisation FNO – a fund that stimulates and supports initiatives improving the opportunities of vulnerable people – developed the program Care and Future Prospects (CFP). The goal of this program was to improve the social position of young people aged 0–25 with a physical or mental chronic condition in five areas: care, school, work, sport, and personal strength. To achieve this aim, forty-five research projects and innovation projects were funded. Thirty-three of these projects were funded during four open calls; twelve received specified funding (not in an open call). Output from the projects included, for example, a tool to improve self-management and a digital platform about performing physical activity with a disability.	
An important element of the program was PPI of young people with a chronic condition. A participatory youth panel was set up with young people with different chronic conditions, such as diabetes, cerebral palsy and traumatic brain injury. All research and innovation projects that received funding were also asked to involve individual young people with a chronic condition that was relevant to the project. For example, projects about young people with rheumatism were asked to work together with young people with rheumatism. Later on in the program, PPI became mandatory for the projects that received funding. Project teams were allowed to decide for themselves, preferably in consultation with the young people, how they would shape the PPI in their project. Within the program, meetings were organised for project teams to share experiences and facilitate PPI.	
Research on the youth panel has been reported elsewhere [25]. The current article focuses on the lessons learned from the PPI of individual young people in the research and innovation projects.	

## Methods

### PPI in the current study

Young people with a chronic condition were actively involved in conducting the overall evaluation study of CFP and in writing this article. During the evaluation study, we regularly spoke to the chair of CFP’s participatory youth panel to discuss draft versions of data collection methods and to reflect on our findings. Other young people with a chronic condition pilot tested the questionnaire to make sure the questions were relevant and comprehensible. A data analysis meeting with young people with a chronic condition and their parents was organised to analyse and interpret the findings of the Involvement Matrix.

This article is a co-production of researchers and experience experts as well. Two young people with a chronic condition are co-authors on this article. They provided critical feedback on draft versions of the article and advised on the practical tips.

### Data collection

The lessons learned in this article are based on four data sources from the overall evaluation study of CFP. One data source provided information from the perspective of young people with a chronic condition, i.e. questionnaires. Three data sources contained information on PPI from the perspectives of project teams, i.e. project reports, case studies of projects and Involvement Matrices. The data we have used concerned 33 research and innovation projects. For most of the projects, we have information from multiple data sources (Table 2).

**Table 2:** Data sources available for projects included in this study

	Number of projects
All four data sources	1
Project report, case study and questionnaires	1
Project report, Involvement Matrix and questionnaires	6
Project report and case study	2
Project report and Involvement Matrix	7
Project report and questionnaires	8
Involvement Matrix and questionnaires	1
Project report	6
Questionnaires	1
**Total**	**33**

#### Questionnaires

To evaluate the projects from the perspective of the young people, a questionnaire has been developed. The questionnaire contained open- and closed-ended questions about the following topics: background, opinion about the content of the project, PPI and outcomes of the project, For the current study, we only used data from the questionnaires addressing PPI of young people with a chronic condition, e.g. what did they do in the project and how much they did do. An example of a question is: ‘How important were you for the project?’. The part of the questionnaires that contained relevant questions for the current study can be found in the supplementary materials.

All project teams were asked to invite the young people who were in any way involved in their project to fill out an online or written version of the questionnaire. Nineteen of the 45 projects co-operated. Young people were invited to fill out the questionnaires within 1 month after the project ended. Per project, the maximum amount of filled out questionnaires was 50. Within the 19 projects, 222 young people filled out the questionnaire (Table 3). The number of questionnaires varied between 1 and 46 per project.

**Table 3:** Characteristics of the young people with a chronic condition who participated in the questionnaire (*N* = 222)

	N	%
**Sex**		
Boy	100	45
Girl	122	55
**Age**		
18 and younger	88	40
19–24	58	27
25–30	59	27
31 and older	15	6
**Living situation**		
With parents	139	63
On their own	26	12
With partner	25	11
In a group	22	10
Different	10	4
**Education or work status**		
Elementary school	3	1
Secondary education	69	31
Post-secondary education	47	22
Unemployed / seeking a job	16	7
Paid employment	49	22
Volunteer work	12	6
Different	25	11

#### Project reports

As a subsidy condition, project teams were requested to fill out a project report at the end of the project. The format of the report contained open- and closed-ended questions, addressing the following topics: general project information, PPI, future of the project, and project outcomes. For the current study, we only used data from the reports addressing PPI of young people with a chronic condition, e.g. how where they involved and in what project phases. A closed-ended question on this topic included, for example, ‘On a scale from 0 to 10, to what extent did the participation of young people yield the desired results?’ The part of the format of the project reports that contained questions about PPI can be found in the supplementary materials. A total of 31 project teams wrote a project report.

#### Case study

Some of the projects were studied in an in-depth case study. Cases were selected in consultation with the CFP bureau. Attention was paid to selecting cases that could provide valuable lessons and that varied in terms of the young people targeted (e.g. different ages and addressing different chronic conditions) and type of project (research or innovation). Five case studies contained information on the PPI of young people with a chronic condition. The characteristics of projects used as case studies are displayed in Table 4.

**Table 4:** Characteristics of case studies and number of project team members interviewed

Case	Goal	Young people addressed	Type^**a**^	Number of project team members interviewed
1	Help students to study and provide them with the opportunity to have a normal student life	Young people with a severe physical impairment (age 18–25)	Innovation	2
2	Develop a tool that enables the active involvement of young people in their care	Young people with a chronic condition in general (age 4–18)	Research	2
3	Provide practical tips on how to participate in society, despite the – sometimes unanticipated - consequences of a chronic condition	Young people with a chronic condition in general (age 12–25)	Research	4
4	Improve the transition of young people with a disability from school to work	Young people following Intermediate Vocational Education (age 16–20)	Research	2
5	Inform students with a functional disability about studying with a disability	(Future) students with a physical impairment (age 15–25)	Innovation	5

^a^ Research projects are scientific projects aimed at increasing knowledge. Innovation projects are practice-oriented projects aimed at developing, for example, tools and interventions

The CFP bureau invited project teams to participate in an interview. In total, 15 members of project teams participated. The interviews were semi-structured using a topic list. Topics included project development, collaboration with other parties, PPI, and lessons learned. For the current study, we used the data on young people’s PPI. For example, questions about this topic included ‘How did you plan to involve young people with a chronic condition, and did the involvement in your project turn out as you planned?’ and ‘How did young people’s PPI influence your project?’

All interviews have been recorded. Based on the recordings, we have written five case descriptions. All case descriptions contained the following sections: the project and its aim, collaboration between project team members, with the funder, with external partners and with young people with a chronic condition and PPI, For the current study, we have used the information in the sections on collaboration with young people with a chronic condition and PPI for further analyses.

#### Involvement matrices

We have used the Involvement Matrix to gain insight into how young people were involved in projects [26]. Originally, the intention of this tool is to facilitate PPI and the dialogue between patients and project teams about their roles in a project [27]. In the current study, we asked project teams to fill out the Involvement Matrix after the project ended.

The Involvement Matrix helps to map PPI activities during different project phases. Project phases are on the vertical axes of the Involvement Matrix, consisting of ‘preparation’, ‘execution’ and ‘implementation’. The roles young people with a chronic condition can play are on the right axe, including ‘listener’, ‘co-thinker’, ‘advisor’, ‘partner’, and ‘decision-maker’ (Table 5). By combining the phases and roles, a matrix with cells is generated. In the current research, we have asked project teams to describe PPI activities that took place, and report these in the corresponding cells.

**Table 5:** Distinction between different roles in the Involvement Matrix

The distinction between different roles in the Involvement Matrix is as follows [26]:	
• *Listeners* are given information about the project by the project team;	
• *Co-thinkers* are asked to give their opinion. Project teams can decide whether they will use it and how;	
• *Advisors* provide solicited and unsolicited advice. Solicited advice is binding. Unsolicited advice must be given formal attention and may only be rejected with substantiated arguments;	
• *Partners* collaborate with other project team members as equals.	
• *Decision-makers* take initiative themselves and/or make necessary decisions.	

Projects teams were invited to fill out an Involvement Matrix. In addition, they were asked to answer some open-ended questions regarding motivations for choosing specific involvement activities and what they perceive as required for meaningful involvement. For some project teams filling out the Involvement Matrix was optional and for others it was compulsory, depending on the subsidy conditions.

Fifteen project teams filled out the Involvement Matrix. All data collected with the Involvement Matrices concerned PPI and were used for further analyses.

### Analysis

All data from the project reports, case studies, Involvement Matrices and questionnaires that concerned PPI were included in the analyses. First, the data from each data source were analysed separately. We quantitatively analysed the data from the project reports and questionnaires in Stata/SE 15, using descriptive statistics. The data from the Involvement Matrices were analysed both quantitatively and qualitatively. We counted the number of projects that reported activities in each cell of the matrix. The open-ended questions were analysed qualitatively in MaxQDA, using thematic analyses. Themes included facilitating factors, impeding factors, designing PPI, positive impact, support and collaboration with young people. For the case studies, we used the case descriptions that were written based on the interview data. In these case descriptions, we highlighted all passages that addressed PPI.

The second step was to conduct an overall analysis to combine the four data sources and extract the lessons learned. We uploaded all findings from the four data sources in MaxQDA. Then, we conducted a thematic analysis to identify themes across the data sources. In other words, we grouped the findings of the different data sources (both qualitative and quantitative) that addressed the same issue. This resulted in six themes: practicalities, involvement from the start, roles and responsibilities, support, flexibility and an open mind and evaluation of process and outcomes. Those themes and the lessons we have learned within the themes are described in the Results section.

### Reporting

We have used the GRIPP2-checklist (Guidance for Reporting Involvement of Patients and the Public) as a guideline in reporting our study [28].

## Results

In this section, we describe the themes from the overall analysis and the lessons learned within these themes about PPI of young people with a chronic condition in research and innovation projects. The lessons are presented following the chronological course of projects; from ‘Practicalities to take into account at the start’ to ‘Evaluation of process and outcomes’.

### Practicalities to take into account at the start

In the development and planning of a participatory project, it is vital to take some practicalities into account.

First, we found that participatory projects demand more time compared to non-participatory projects. In the project reports, case study and Involvement Matrices, project teams and coordinators noted that young people’s PPI frequently – if not consistently – had consequences for the continuity of projects. For example, one project coordinator added the following statement to her Involvement Matrix: ‘Before you start, you have to clearly plan the moments you will talk to young people, so you actually have to make time to think about this’. One of the cases in the case study provided a good example of how PPI can be time consuming. This case concerned an innovation project aimed at improving information provision for future students with disabilities. The planning in this project regularly had to be adapted to the needs of the young people involved. The project coordinator noted, for example, that the young people involved preferred individual meetings with the project coordinator, instead of the planned group meetings. Changing this took time, and caused the project team to postpone deadlines in the planning. According to the project coordinator, events like this highlight the need for a flexible planning in project plans and proposals.

From the case study and Involvement Matrices, we have learned that the reimbursement of young people is another practicality to consider, and should also be included in project plans and proposals. Reimbursements are a valuable and tangible appreciation for young people’s input. Moreover, reimbursing young people increases their commitment to their tasks and the project, and thereby clarifies mutual expectations about roles and responsibilities. A project coordinator explained in the Involvement Matrix: ‘It is essential to give them a compensation for their efforts. […] That is most certainly an encouragement, but even then it is difficult to involve young people and keep them engaged’. In most projects, young people received gift vouchers for involvement efforts. This is a relatively easy way to reimburse young people. However, a minority of the project coordinators chose to offer them a paid appointment in their organisation, since they believed this could facilitate involvement as equal partners.

In one of the cases in the case study, the project team extensively described the process of providing young people with rheumatism with a paid appointment in a hospital. They came up with two recommendations. The first one was to let young people themselves decide how they wish to be appointed, for example with a permanent contract, a zero-hours contract, or as a freelancer with a specific assignment. This is important, because some forms of reimbursements can interfere with the disability benefits young people with a chronic condition may receive. The project team can provide information on pros and cons of each possibility, so young people can decide what fits them and their situation best. Their second recommendation was to make sure that working conditions are adapted to the needs of the young people involved. For example, in their project, they needed to make sure workplaces were accessible by elevator. They also adapted the working hours, as the unexpected course of rheumatism required flexibility.

A third practicality in developing and planning a participatory project is the recruitment of young people with a chronic condition and gaining their commitment. This appears to be a difficult task, since not all young people wish to be engaged in matters that relate to their condition, and project teams do not know where to find them. Of all project coordinators who filled out a project report, one in five mentioned the vulnerability and therefore the limited input of young people with a chronic condition as a factor that impeded the progress of their project. From the case study, we learned that collaborating with patient or youth organisations and developing participatory activities that appeal to young people are facilitating factors in recruitment. For example, in a research project, meetings of its young people’s advisory board were organised in a Dutch theme park. After the meetings, young people were given free entry to the theme park.

**Table:** 

**Practical Tips**	
**For young people**	**For project teams**
► If you wish to be involved in a project, let others know. A patient or youth organisation may be able to help you.	► PPI takes time. Take this into account and set up a flexible planning schedule.
► If possible, consult with other young people who are involved in the project about how they handle their involvement.	► Reward young people for their involvement. Think about how you wish to reimburse them and how you deal with expenses they have to make, such as travelling costs. Discuss the possibilities with them and make the necessary arrangements.
► Agree on how you will be rewarded for your involvement and how you deal with expenses you have to make, such as travelling costs.	► Rewards for PPI may interfere with disability benefits young people receive. Take this into account, when discussing the possibilities.
► Make sure you have sufficient time to play a meaningful role in the project.	► Make sure you timely start recruiting young people. Collaborating with a patient or youth organisation may be helpful.

### Involvement from the start

For an effective PPI process, it is advantageous to collaborate with young people with a chronic condition from the start of a project. Involving them in generating new project ideas and in writing project proposals can contribute to the meaningfulness of their involvement. In the case study and Involvement Matrices, project teams and coordinators have noted that this allows young people with a chronic condition to influence the content and course of the project, before it is set in stone. Also, it facilitates the collaboration between the young experience experts and other members of the project team as equal partners. A project coordinator who filled out the Involvement Matrix: ‘One adolescent with a developmental language disorder thought with us about the content of the project, before it even started. From the start of the project, he was involved as a paid employee. This contributed to the equal collaboration between him and the rest of the project team’.

In CFP, some projects were initiated by young people with a chronic condition and, as a result, they were inherently involved from an early stage. In these projects, the relevance for and interest of young people was guaranteed. However, most project ideas did not come from young people with a chronic condition. In these cases, both the case study and the Involvement Matrices show that project teams should actively seek for the input of experience experts in generating ideas and writing a proposal. In one of the cases in the case study, concerning a research project that addressed the (psycho)social consequences of having a chronic condition, the research question was formulated based on the experiences of young people. In collaboration with other members of the project team, experience experts conducted interviews to explore issues that matter to young people with a chronic condition and translated these into a relevant research question. Other ways to involve young people with a chronic condition from the start are by asking them to co-write a project proposal or to provide feedback on draft versions.

**Table:** 

**Practical Tips**	
**For young people**	**For project teams**
► If possible, co-write the project proposal. Introduce your experiences and ideas.	► Invite young people to contribute to or co-write the project proposal.
	► Think about how you will obtain the resources you need to involve young people in writing a project proposal, such as time and reimbursements, as funding may not yet be available during this phase.

### Roles and responsibilities

To facilitate commitment and meaningful PPI of young people, it is necessary to create a good and equal partnership between them and the other members of the project team. Becoming a successful team is dependent on taking young people seriously, listening to them, and providing them with a certain amount of autonomy. A project coordinator who filled out the Involvement Matrix reported: ‘Don’t just pretend you are listening, but actually follow-up on their advices’.

From the case study and Involvement Matrices, we have learned that an essential step in creating a partnership is defining roles and responsibilities. Clarity about roles and responsibilities helps young people to understand what is expected from them and what their contribution is to the project. As such, project teams argued that PPI activities should be concrete and well-defined. For example, a project coordinator noted in the Involvement Matrix: ‘Young people need clarity about what is expected from them. Therefore, as a project coordinator, it was important to make clear arrangements.’

From the questionnaires and Involvement Matrices, we learned that young people with a chronic condition and project teams can have different perspectives on roles and responsibilities. Table 6 shows the results of the questionnaire among young people with a chronic condition. There is variation in how they perceive their roles in the projects. They see themselves mainly as participants in projects that were set up for them. They also found they were mostly passively involved, by receiving information. Smaller percentages of young people reported giving their opinion, cooperating in the execution of a project, thinking about (further) improvements, generating publicity, or cooperating on the design of the project. They were the least involved in helping with recruitment.

**Table 6:** Roles young people with a chronic condition played in projects, based on the perceptions of young people involved in nineteen different projects (multiple answers possible; *N* = 217)

I…	N	%
… was one of the (passive) participants	170	78
… received information about the project	70	32
… gave my opinion about the project	47	22
… cooperated on the execution of the project	47	22
… thought about (further) improvements of the project	40	18
… cooperated on the design of the project	34	16
… generated publicity for the project	26	12
… helped recruiting participants for the project	19	9

When we asked young people in the questionnaire (*N* = 226) to quantify how much they did in the project they were involved in, 5% reported they did ‘really a lot’ and 18% did ‘a lot’. Approximately a third reported they did ‘pretty much’ (38%). The remaining young people answered they did ‘a little’ (35%) or ‘nothing at all’ (4%).

Comparing this to the perspectives of the other members of the project teams, we see that project teams reported a more active role of young people. According to the results of the Involvement Matrices, young people were generally involved in all project phases, e.g. preparation, execution and implementation. They were mostly co-thinkers. In this role, they provided feedback on a training developed by other members of the project team or on the items in a concept questionnaire. It must be noted that some project teams classified young people with a chronic condition co-thinkers, when they were actually study respondents sharing their experiences through interviews or questionnaires. These cases were removed from Table 7. In CFP projects, young people were also regularly advisors and partners. They advised project teams on the development of, for example, a book, game or training. Partners participated by (co-)writing blogs, (co-)presenting at conferences and (co-)organising trainings. The roles at the extremes of the spectrum, e.g. ‘listener’ and ‘director’, were the least often reported. Listeners were mostly informed by the project teams about the progress of the project or future participatory activities. In the role of director, young people autonomously carried out trainings or filmed and interviewed professionals in the field.

**Table 7:** Roles young people with a chronic condition played in different phases of projects according to the project teams (*N* = 15)

N	Listener	Co-thinker	Advisor	Partner	Decision-maker	Total^**a**^
**Total**^**a**^	8	13	10	10	6	x
**Preparation**	3	8	6	5	0	13
**Execution**	5	9	6	7	6	14
**Implementation**	2	9	7	4	3	15

^a^ The number of unique projects that involved young people with a chronic condition in a specific role or during a specific phase. The totals are lower than the sum of the rows and columns, since young people with a chronic condition can play multiple roles during one phase

In general, project teams did not (explicitly) describe why young people were involved in a specific role or phase. However, the open-ended questions of the Involvement Matrix showed that project teams not necessarily strive to provide young people with as much influence as possible. According to them, key in achieving a good and equal partnership is that PPI activities match the experiences and abilities of young people with a chronic condition, and cannot be too strenuous. Several project coordinators recommended involving young people in determining what their responsibilities will be, and not deciding this for them. A project coordinator who reported limited PPI in a project noted: ‘If we want to involve young people with a chronic condition to a greater extent, we have to involve them from the start, so they can indicate themselves how they can and wish to be involved’. Together, they can discuss what young people wish to do, how much time and energy they have, and choose fitting activities.

In one of the cases in the case study, the project team explicitly highlighted the importance of involving young people in determining their roles and responsibilities. In their project, they noticed that the young people involved were not always able to follow up on agreements. The enthusiasm of these young people seemed to be overshadowed by their limited energy. Formulating clear activities helped them to focus on specific tasks, and accomplish these.

**Table:** 

**Practical Tips**	
**For young people**	**For project teams**
► Discuss with the project team what you can and want to do and what you need for this. Ask, for example: ‘Where and when do we meet and what do I have to do, exactly?’.	► Listen to what young people have to say. Only ask for their involvement, when you can follow-up on their opinions and feedback.
► Regularly discuss whether your input and role are still clear to you and the other members of the project team.	► Involve young people in determining their roles and responsibilities.
► If – for whatever reason – you cannot complete the tasks you agreed to do, be honest about it. Together with the project team you can find a solution for this.	► Keep tasks of young people clear. Discuss tasks and responsibilities of other members of the project team with them as well.
► You can use the Involvement Matrix as a tool in discussing your roles and responsibilities before and during the project.	► Regularly discuss with young people how they are doing. If necessary, adapt their roles and responsibilities.
	► You can use the Involvement Matrix as a tool in discussing young people’s roles and responsibilities before and during the project.

### Support

In the project reports, one in five project coordinators reported the vulnerability of young people with a chronic condition as a factor that impeded the progress of their project. According to the findings of the case study and the Involvement Matrices, this vulnerability sometimes limited possibilities for involvement. Due their chronic condition, the young people frequently had to deal with, for example, lower energy levels, health issues or sudden hospital stays. According to a project coordinator who filled out the Involvement Matrix, it is important that ‘young people can shape and carry out the project process in their own way and within the restrictions of their time and energy’. When young people drop out, additional time has to be invested in finding other young people who are willing to be involved. Attention should be paid to the fact that young people with a chronic condition have to perform PPI activities within the restrictions of their (limited) energy.

The vulnerability of young people with a chronic condition highlights the importance of providing sufficient support. One project coordinator who filled out the Involvement Matrix reported that ‘it is necessary that the right professionals support and coach young people from the start’. Project leaders state that training and coaching can make PPI less strenuous, as it improves the match between PPI activities and the knowledge and skills of young people. Project teams can also benefit from training and coaching. The project coordinators did not specify what topics should be addressed in training and coaching for young people with a chronic condition and project teams.

**Table:** 

**Practical Tips**	
**For young people**	**For project teams**
► Be open about your knowledge and skills and your physical and mental capacities. The project team will keep this in mind.	► Be sensitive to the capacities and possibilities of all members of the project team and take this into account. If necessary, organise training and coaching.
► Determine for yourself what you need to perform your tasks. Ask for help if you need it and be clear about what working conditions suit your needs.	► Discuss with the young people how they would like to meet. Be aware that travelling may be an obstacle to some. Also consider conducting meetings online or by phone.

### Flexibility and an open mind

Experiences reported in the case study and Involvement Matrices taught us that flexibility and an open mind are essential for meaningful PPI. In all project phases, young people need to be provided with ‘space’ to make a difference. That is to say, project teams have to make time to listen attentively to the ideas of young people, and take them seriously. Furthermore, a willingness to change initial project plans is fundamental for the input of young people to lead to real and meaningful changes in the project. A project coordinator who filled out the Involvement Matrix: ‘[For meaningful PPI] you need the ability to process feedback and to do some things completely different than you planned’.

The importance of flexibility and an open mind was especially noted in one of the cases in the case study about an innovation project aimed at improving information provision for future students with disabilities. The project team experienced that participatory processes never develop as expected, and found it difficult – if not impossible – to determine in advance how project goals would be achieved. In the project, students were invited to make vlogs about their student life, and, initially, they were enthusiastic and eager to participate. However, during the project, it became clear that some young people found it too confronting to film themselves, causing them to drop-out. To prevent the project from failing, the project team decided to make several adaptations to the initial project plans. For example, instead of filming themselves, they invited young people to an interview on camera or to write their experiences down on paper. Also, when young people did not want to share their experiences, they were asked to be involved in technical activities, such as filming or editing.

The revisions of the initial project plans facilitated the PPI of young people. According to the project team, it contributed to the involvement of more young people in making the videos than would have been the case if the initial plans were followed. Consequently, they concluded that only project results should be determined in advance; the processes leading to these results should be flexible. During the project, the project team should ‘be bold and move along’ with the young people, and jointly discover the best way to achieve project results.

**Table:** 

**Practical Tips**	
**For young people**	**For project teams**
► Every member of the project team is important. Be open to input of other’s.	► Take time to listen attentively to the ideas of young people and take them seriously.
	► Be flexible and willing to change initial project plans. You don’t know in advance what the input of members of the project team, and especially the young people, will be.

### Evaluation of process and outcomes

During and after a participatory project, it is important to critically evaluate processes and outcomes. This can provide valuable feedback to both young people with a chronic condition and the other members of the project team. It also contributes to the motivation of the people involved. In the open-ended questions of the Involvement Matrix, one of the project teams reported that young people find it valuable to know how their advices are used in practice. In addition, in another project where parents were involved on behalf of their children, the project coordinator noticed: ‘it is more meaningful for parents to participate, when the project has a tangible, positive impact on their child’.

In our evaluation, we also looked at the impact of young people’s PPI. We learned that both young people with a chronic condition and other members of project teams experience a positive impact of the PPI of young people with a chronic condition on the projects. In the questionnaire, more than two out of five young people experienced they were ‘(really) important’ to the project (Table 8). They valued their own contributions, since they shared their experiential knowledge, participated in meetings that helped others, and helped to set up projects and disseminate project results. Project coordinators rated the extent to which the PPI of young people with a chronic condition yielded the desired results in the project reports on average an 8 on a scale from 0 to 10. They described young people’s contribution as ‘unique’ and ‘irreplaceable’. According to the project teams, young people improved the relevance and quality of their projects.

**Table 8:** Extent to which young people with a chronic condition were important to projects, based on the perceptions of young people involved in nineteen projects (*N* = 226)

Extent to which they considered themselves important to the project	%
Really important	9
Important	34
A little important	23
Not important at all	3
Doesn’t know	31

Despite the enthusiasm about the value of young people’s PPI, it seemed to be difficult to describe the specific impact of their input in the project reports, case study and Involvement Matrices. Project coordinators mostly provided general descriptions of impact, such as ‘without their [young people’s] input, the project would not have been of the same quality’. Concrete contributions mentioned by project coordinators include that young people can facilitate the recruitment of other young people, that they can assess the relevance of projects, and that they can help disseminate project findings. However, we have gained little insight in the extent to which young people’s PPI contributed to the actual outcomes of a project; project teams were unable to specify this.

**Table:** 

**Practical Tips**	
**For young people**	**For project teams**
► Try to learn from feedback. Ask for feedback yourself.	► Provide young people with feedback on what they do and how they do it, so they can learn from it.
► Your experiences are important for the project, so don’t be afraid to say what you want to say! The project team can learn from your feedback.	► Critically and systematically evaluate the involvement process and its outcomes. Come up with an evaluation plan in advance, and evaluate both the process and outcomes of PPI.

## Discussion

In the current study, we aimed to discuss the lessons we learned about PPI of young people with a chronic condition, drawn from a case study of a program that hosted numerous participatory projects over a period of 4 years. These lessons contribute to the existing evidence base of young people’s PPI by providing practical examples and tips on how young people with a chronic condition and adult members of project teams can work together, what difficulties they can encounter, and what is needed for meaningful PPI.

In part, our findings echo the results of previous studies. It is, for example, well-known that projects in which young people with a chronic condition are involved take more time than non-participatory projects [4–6, 15] and previous studies have also emphasised the importance of involving them in the early phases of a project [5, 29, 30]. The importance of reimbursements [4, 29, 30] and support [15, 17, 31, 32] has been reported in previous studies as well. Our study adds to this knowledge by providing practical examples of these challenges and – more importantly – of how these challenges can be addressed. Moreover, we have taken it one step further by formulating practical tips that are helpful in organising a participatory project.

The lessons we have presented concern both the *process* and *outcomes* or *impact* of involving young people with a chronic condition in projects. The lessons learned about the process have shown that meaningful PPI starts with – or even before – the writing of the project proposal and continues all the way through the dissemination of the results. During the entire project, time and resources need to be invested in the collaboration. This involved continuous discussions on the roles of young people with a chronic condition and other members of project teams and the development of the project, and whether these are (still) aligned with everyone’s needs and preferences.

Regarding the *outcomes* or *impact* of PPI of young people with a chronic condition, we have noted a general enthusiasm about the value of working together. From the literature, we know that young people’s PPI can have positive impacts on, for example, the design of projects [4, 19], the people involved [4, 15, 19, 32], and the dissemination of project results [4–7, 19]. However, the evidence of this impact is of relatively low quality [2]. In the current article, we also found that project teams had a hard time specifying the impact of PPI, as became clear from the relatively few and vague examples they provided. Approximately a third of the young people were unable to specify how important they were to a project. PPI is a complex process and therefore it is difficult to exactly determine its impact [2, 33, 34]. This highlights the need for research on how to evaluate the impact of PPI with young people with a chronic condition. As has become clear from our results, knowing that working together has a (positive) impact can motivate young people with a chronic condition and other members of the project team and teach them about how they should work together.

### PPI in the current study

In conducting this study and writing the manuscript, researchers have worked together with experience experts. In general, this enabled us to match data collection methods to the young people who had to fill them out and to reflect on our findings and place them in the right context. For example, the pilot tests of the questionnaire helped us to simplify questions and answering possibilities. The analysis of the Involvement Matrices with young people taught us that analysing open-ended questions is difficult for young people who are not researchers and who have no experience with studying other people’s opinion and experiences. They emphasised their own experiences over the data. Some chose to not include some statements of project coordinators, because they did not agree with him or her. Although we were not able to use the analyses made by those young people, they did provide insight in differences in perspectives between project coordinators and young people.

Co-producing this manuscript with experience experts has been a valuable experience. Reflecting on their PPI, the experience experts explained that they considered this to be a valuable experience as they were involved from the start of the writing process. This allowed them to not only reflect and agree on what the researchers wrote, but they were also able to co-write from the start. As a result, they felt their contribution to be valuable and appreciated. They stated: ‘In our opinion, it is very important to involve young people as much as possible in research and policy that concerns them. However, as the time and energy of researchers is precious, so is ours. We would like to contribute, but only if and when we can be of value.’ The researchers and experience experts who wrote this manuscript agree that their collaboration enabled them to describe lessons learned and practical tips that are understandable and relevant to both young people with a chronic condition and project teams.

### Strengths and limitations

The current study had some limitations. First, the majority of the data sources we used were based on the experiences of project teams. Solely the questionnaire focused on perspectives of young people and not all projects participated in the questionnaire. Consequently, most results are based on project teams’ perspectives. We recommend that future research includes young people’s perspectives to a greater extent. Second, we invited all young people who were involved in projects in any way to fill out the questionnaire; also those who played a passive roles, e.g. receivers of an intervention or users of a tool created in the project. This explains the relatively large part of young people reporting they were (passive) participants in their project. Ideally, we would only have reported findings for the young people who (should have) played a PPI role, but we were unable to make this distinction. Finally, there is a risk of response bias in the data sources. The project reports of the project coordinators, for example, were not solely used for our research, but also for reporting progress to the funder (FNO). Furthermore, in the case of the Involvement Matrix, not all project coordinators were obliged to fill out the Involvement Matrix. This may have resulted in selection effects; project coordinators who wanted to fill out the Involvement Matrix may have been more positive towards young people’s involvement compared to project coordinators who did not. The young people with a chronic condition who returned the questionnaire may also have been more positive attitude towards their PPI in a project than the non-responders, as they are likely to be more eager to share their contributions to the project. However, in the project reports, the Involvement Matrix and the questionnaires, project coordinators and young people were critical of the PPI in their project. Project coordinators provided critical descriptions of involvement in their project, suggesting they were eager to learn from both the facilitators and barriers they experienced. Also, a considerable part of the young people who participated in the questionnaires consider themselves only a little or not important to a project, suggesting they were critical as well.

An important strength of this study is its size. The lessons learned are drawn from a large participatory program that has funded 33 different participatory projects. Also, data were derived using multiple research methods, both qualitative and quantitative.

## Conclusion

In the literature, calls have been made to share experiences and lessons learned on PPI and how to deal with its challenges. The lessons learned in the current study have led us to draw two conclusions. First, meaningful PPI requires effort, time and resources from both young people with a chronic condition and project teams, from the beginning to the end. It is important to continuously discuss roles and responsibilities. Second, we found that the young people and project teams were enthusiastic about their collaboration, but found it difficult to specify its impact. This highlights the need for more research on evaluating the impact of PPI.

The translation of the lessons learned into practical tips is a valuable contribution of this study to the PPI field. The lessons learned provide examples on how others have dealt with challenges in PPI; translating them into practical tips shows young people and project teams what concrete actions they can undertake to achieve successful PPI. As a guide for future PPI projects, a document in which all practical tips are combined has been added to the supplementary materials.

## Supplementary information

**Additional file 1.** Questionnaire for young people with a chronic condition.

**Additional file 2.** Project report format for project coordinators.

**Additional file 3.** Practical tips on Patient and Public Involvement with young people with a chronic condition.

## Data Availability

The datasets used and/or analysed during the current study are available from the corresponding author on reasonable request.

## Abbreviations

PPI: Patient and Public Involvement
CFP: (The program) Care and Future Prospects

## References

[1] INVOLVE (2013). Approaches to public involvement in research.

[2] Van Schelven F, Boeije H, Mariën V, Rademakers J. Patient and public involvement of young people with a chronic condition in projects in health and social care: a scoping review. Health Expect. 10.1111/hex.13069. 10.1111/hex.13069 PMC7495073 32372423

[3] Bailey S, Boddy K, Briscoe S, Morris C (2015). Involving disabled children and young people as partners in research: a systematic review. Child Care Health Dev.

[4] Flicker S (2008). Who benefits from community-based participatory research? A case study of the positive youth project. Health Educ Behav.

[5] Marshall Z (2012). Navigating risks and professional roles: research with lesbian, gay, bisexual, trans, and queer young people with intellectual disabilities. J Empir Res Hum Res Ethics.

[6] Van Staa A, Jedeloo S, Latour JM, Trappenburg MJ (2010). Exciting but exhausting: experiences with participatory research with chronically ill adolescents. Health Expect.

[7] Sloper P, Lightfoot J (2003). Involving disabled and chronically ill children and young people in health service development. Child Care Health Dev.

[8] United Nations General Assembly (1989). Convention on the rights of the child.

[9] Hart RA (1992). Childrens participation: from tokenism to citizenship.

[10] Arnstein S (1969). A ladder of citizen participation. J Am Inst Plann.

[11] Dedding C. Delen in macht en onmacht. Amsterdam: Universiteit van Amsterdam; 2009.

[12] Hart R, Reid A, Bruun Jensen B, Nikel J (2008). Stepping back from the ladder: Reflections on a model of participatory work with children. Participation and learning: perspectives on education and the environment, health and sustainability.

[13] Tritter JQ, McCallum A (2006). The snakes and ladders of user involvement: moving beyond Arnstein. Health Policy.

[14] Shier H (2001). Pathways to participation: openings, opportunities and obligations. Child Soc.

[15] Franklin A, Sloper P (2009). Supporting the participation of disabled children and young people in decision-making. Child Soc.

[16] Graham N, Mandy M, Clarke C, Morriss-Roberts C (2017). Using children and young people as advocates to inform research design. Br J Occup Ther.

[17] Murray R (2012). Sixth sense: the disabled children and young people’s participation project. Child Soc.

[18] Coad JE, Shaw KL (2008). Is children’s choice in health care rhetoric or reality? A scoping review. J Adv Nurs.

[19] Rosen-Reynoso M, Kusminsky M, Gragoudas S, Putney H, Crossman MK, Sinclair J (2010). Youth-based participatory research: lessons learned from a transition research study. Pediatrics.

[20] Kramer J, Barth Y, Curtis K, Livingston K, O'Neil M, Smith Z (2013). Involving youth with disabilities in the development and evaluation of a new advocacy training: project TEAM. Disabil Rehabil.

[21] Lundy L (2018). In defence of tokenism? Implementing children’s right to participate in collective decision-making. Childhood.

[22] Ferguson D, Monfaredi Z, Pussegoda K, Garritty C, Lyddiatt A, Shea B (2018). The prevalence of patient engagement in published trials: a systematic review. Res Involv Engagem.

[23] Ketelaar M, Smits DW, van Meeteren K, Klem M, Alsem M, Imms C, Green D (2020). Involvement of young people and families in all stages of research: what, why and how?. Participation: optimising outcomes in childhood-onset neurodisability.

[24] Hwang W, Weller W, Ireys H, Anderson G (2001). Out-of-pocket medical spending for care of chronic conditions. Health Aff.

[25] Van Schelven F, Boeije H, Inhulsen MI, Sattoe JNT, Rademakers J. “We know what we are talking about”: experiences of young people with a chronic condition involved in a participatory youth panel and their perceived impact. Child Care Pract. 2019. 10.1080/13575279.2019.1680529.

[26] Smits DW, van Meeteren K, Klem M, Alsem M, Ketelaar M. Designing a tool to support patient and public involvement in research projects: the Involvement Matrix. Res Involv Engagem. 2020. 10.1186/s40900-020-00188-4. 10.1186/s40900-020-00188-4 PMC7296703 32550002

[27] Smits DW, Klem M, Ketelaar M (2019). The involvement matrix: involvement of patients in projects and research.

[28] Staniszewska S, Brett J, Simera I, Seers K, Mockford C, Goodlad S (2017). GRIPP2 reporting checklists: tools to improve reporting of patient and public involvement in research. BMJ.

[29] Lightfoot J, Sloper P (2003). Having a say in health: involving young people with a chronic illness or physical disability in local health services development. Child Soc.

[30] Castensoe-Seidenfaden P, Reventlov Husted G, Teilmann G, Hommel E, Olsen BS, Kensing F (2017). Designing a self-management app for young people with type 1 diabetes: methodological challenges, experiences, and recommendations. JMIR Mhealth Uhealth.

[31] Moreau KA, Eady K (2017). The involvement of adolescents with chronic health conditions in medical education: an exploratory qualitative study. Hosp Pediatr.

[32] Stevenson M (2014). Participatory data analysis alongside co-researchers who have Down syndrome. J Appl Res Intellect Disabil.

[33] Greenhalgh T, Hinton L, Finlay T, Macfarlane A, Fahy N, Clyde B, Chant A (2019). Frameworks for supporting patient and public involvement in research: systematic review and co-design pilot. Health Expect.

[34] Collins M, Long R, Page A, Popay J, Lobban F (2018). Using the public involvement impact assessment framework to assess the impact of public involvement in a mental health research context: a reflective case study. Health Expect.

