# Supplementary material for: Patient and public involvement of young people with a chronic condition: lessons learned and practical tips from a large participatory program
Source: Res Involv Engagem. 2020 Sep 30;6:59. doi: 10.1186/s40900-020-00234-1 (PMC7525958; doi:10.1186/s40900-020-00234-1)
Supplement: Supplementary file 1 — Additional file 1. Questionnaire for young people with a chronic condition. [file 40900_2020_234_MOESM1_ESM.docx]

**Questionnaire for young people with a chronic condition**

**Background**

1. **How old are you?**
   ……………… years old
2. **Are you a boy or a girl?**□ Boy
   □ Girl
3. **What situation is most applicable to you**□ I am in elementary school
   □ I am in secondary education

□ I am in post-secondary education
□ I am unemployed / looking for a job
□ I have a paid job
□ I volunteer
□ Other

1. **How do you live?**
   □ I live at my parents’ house
   □ I live by myself, without other people

□ I live by myself, with other people in one house
□ I live with a friend
□ I live in a care facility
□ I volunteer
□ Other

**Questions about the project**

1. **What did you do in the project?**
   □ I received information about the project
   □ I gave my opinion about the project

□ I was ambassador
□ I was involved in the design of the project
□ I was involved in the execution of the project
□ I helped finding participants for the project

□ I was one of the participants myself
□ I was involved in thinking about how the project can be improved

□ I was involved in making sure that others know and use the project
□ Other

1. **How much did you do in the project?**
   □ Nothing at all
   □ A little

□ Pretty much
□ A lot
□ Really a lot

1. **How important were you to the project?**
   □ Not important at all
   □ A little important

□ I don’t know
□ Important
□ Very important

1. **If you want to, you can explain your answer here:**
   ………………………………………………………………………………………………………………………………………………………………………………………………………………………………………………………………………………………………………………………………………………………………………………………………………………………………………………………………………………………………………………………………………………………………………………………………………………………………………………………………………………………
