# Supplementary material for: Patient and public involvement of young people with a chronic condition: lessons learned and practical tips from a large participatory program
Source: Res Involv Engagem. 2020 Sep 30;6:59. doi: 10.1186/s40900-020-00234-1 (PMC7525958; doi:10.1186/s40900-020-00234-1)
Supplement: Supplementary file 2 — Additional file 2. Project report format for project coordinators. [file 40900_2020_234_MOESM2_ESM.docx]

**Project report format for project coordinators**

**Questions about involvement of young people with a chronic condition**

1. **To what extent did you involve young people with a chronic condition in the project?**

| 0 | 1 | 2 | 3 | 4 | 5 | 6 | 7 | 8 | 9 | 10 |
| --- | --- | --- | --- | --- | --- | --- | --- | --- | --- | --- |
| □ | □ | □ | □ | □ | □ | □ | □ | □ | □ | □ |
| Not at all | |  |  |  |  |  |  |  | In full | |

1. **How where young people with a chronic condition involved in your project?***Multiple answers possible*
   □ We informed them
   □ We consulted them

□ They advised us
□ They designed and executed the projects together with us
□ They co-decided during the project
□ Other
□ Not applicable, young people were not involved in our project

1. **During what phase of the project did you involve young people with a chronic condition?**□ During the development of first ideas for the project
   □ During writing and submitting the project proposal
   □ During the design of the project
   □ During the execution of the project
   □ During the dissemination and implementation of project results
   □ During the evaluation of the project
   □ Other
   □ Not applicable, young people were not involved in our project
2. **To what extent did the involvement of young people with a chronic condition yield the desired results?**

| 0 | 1 | 2 | 3 | 4 | 5 | 6 | 7 | 8 | 9 | 10 |
| --- | --- | --- | --- | --- | --- | --- | --- | --- | --- | --- |
| □ | □ | □ | □ | □ | □ | □ | □ | □ | □ | □ |
| Not at all | |  |  |  |  |  |  |  | In full | |

**Can you explain your answer?**

………………………………………………………………………………………………………………………………………………………………………………………………………………………………………………………………………………………………………………………………………………………………………………………………………………………………………………………………………………………………………………………………………………………………………………………………………………………………………………………………………………………
