# Supplementary material for: Patient and public involvement of young people with a chronic condition: lessons learned and practical tips from a large participatory program
Source: Res Involv Engagem. 2020 Sep 30;6:59. doi: 10.1186/s40900-020-00234-1 (PMC7525958; doi:10.1186/s40900-020-00234-1)
Supplement: Supplementary file 3 — Additional file 3. Practical tips on Patient and Public Involvement with young people with a chronic condition. [file 40900_2020_234_MOESM3_ESM.docx]

**Practical tips on Patient and Public Involvement with young people with a chronic condition**

Young people with a chronic condition are increasingly involved in doing research and developing tools and interventions that concern them. Working together with patients is called Patient and Public Involvement (PPI). We know from the literature that PPI with young people with a chronic condition can be challenging. In the article ‘Patient and Public Involvement of young people with a chronic condition: Lessons learned and practical tips from a large participatory program’, we have formulated tips that can help in achieving successful PPI. In this document, we provide an oversight of all practical tips for young people and for project teams.

| **Practicalities to take into account at the start** | | | |
| --- | --- | --- | --- |
|  | **Tips for young people** |  | **Tips for project teams** |
| ►  ►  ►  ► | If you wish to be involved in a project, let others know. A patient or youth organisation may be able to help you.  If possible, consult with other young people who are involved in the project about how they handle their involvement.  Agree on how you will be rewarded for your involvement and how you deal with expenses you have to make, such as travelling costs.  Make sure you have sufficient time to play a meaningful role in the project. | ►  ►  ►  ► | PPI takes time. Take this into account and set up a flexible planning schedule.  Reward young people for their involvement. Think about how you wish to reimburse them and how you deal with expenses they have to make, such as travelling costs. Discuss the possibilities with them and make the necessary arrangements.  Rewards for PPI may interfere with disability benefits young people receive. Take this into account, when discussing the possibilities.  Make sure you timely start recruiting young people. Collaborating with a patient or youth organisation may be helpful. |
| **Involvement from the start** | | | |
|  | **Tips for young people** |  | **Tips for project teams** |
| ► | If possible, co-write the project proposal. Introduce your experiences and ideas. | ►  ► | Invite young people to contribute to or co-write the project proposal.  Think about how you will obtain the resources you need to involve young people in writing a project proposal, such as time and reimbursements, as funding may not yet be available during this phase. |
| **Roles and responsibilities** | | | |
|  | **Tips for young people** |  | **Tips for project teams** |
| ►  ►  ►  ► | Discuss with the project team what you can and want to do and what you need for this. Ask, for example: ‘Where and when do we meet and what do I have to do, exactly?’.  Regularly discuss whether your input and role are still clear to you and the other members of the project team.  If – for whatever reason – you cannot complete the tasks you agreed to do, be honest about it. Together with the project team you can find a solution for this.  You can use the Involvement Matrix as a tool in discussing your roles and responsibilities before and during the project. | ►  ►  ►  ►  ► | Listen to what young people have to say. Only ask for their involvement, when you can follow-up on their opinions and feedback.  Involve young people in determining their roles and responsibilities.  Keep tasks of young people clear. Discuss tasks and responsibilities of other members of the project team with them as well.  Regularly discuss with young people how they are doing. If necessary, adapt their roles and responsibilities.  You can use the Involvement Matrix as a tool in discussing young people’s roles and responsibilities before and during the project. |
| **Support** | | | |
|  | **Tips for young people** |  | **Tips for project teams** |
| ►  ► | Be open about your knowledge and skills and your physical and mental capacities. The project team will keep this in mind.  Determine for yourself what you need to perform your tasks. Ask for help if you need it and be clear about what working conditions suit your needs. | ►  ► | Be sensitive to the capacities and possibilities of all members of the project team and take this into account. If necessary, organise training and coaching.  Discuss with the young people how they would like to meet. Be aware that travelling may be an obstacle to some. Also consider conducting meetings online or by phone. |
| **Flexibility and an open mind** | | | |
|  | **Tips for young people** |  | **Tips for project teams** |
| ► | Every member of the project team is important. Be open to input of other’s. | ►  ► | Take time to listen attentively to the ideas of young people and take them seriously.  Be flexible and willing to change initial project plans. You don’t know in advance what the input of members of the project team, and especially the young people, will be. |
| **Evaluation of process and outcomes** | | | |
|  | **Tips for young people** |  | **Tips for project teams** |
| ►  ► | Try to learn from feedback. Ask for feedback yourself.  Your experiences are important for the project, so don’t be afraid to say what you want to say! The project team can learn from your feedback. | ►  ► | Provide young people with feedback on what they do and how they do it, so they can learn from it.  Critically and systematically evaluate the involvement process and its outcomes. Come up with an evaluation plan in advance, and evaluate both the process and outcomes of PPI. |
